# Rapid online analysis of *n*-alkanes in gaseous streams via APCI mass spectrometry

**DOI:** 10.1007/s00216-024-05182-3

**Published:** 2024-02-15

**Authors:** Jonas Wentrup, Ingmar Bösing, Thomas Dülcks, Jorg Thöming

**Affiliations:** 1 Faculty of Production Engineering, Chemical Process Engineering, University of Bremen, Leobener Strasse 6, 28359 Bremen, Germany; 2 Center for Environmental Research and Sustainable Technology, University of Bremen, Postbox 330 440, 28334 Bremen, Germany; 3 FB 02, Mass Spectrometry Service Facility, University of Bremen, Leobener Str. NW2A, 28359 Bremen, Germany; 4 MAPEX Center for Materials and Processes, University of Bremen, Postbox 330 440, 28334 Bremen, Germany

**Keywords:** Gas-phase analysis, APCI mass spectrometry, Hydrocarbons, Fragmentation, Dynamics, Online monitoring

## Abstract

Online monitoring of dynamic chemical processes involving a wide volatility range of hydrocarbon species is challenging due to long chromatographic measurement times. Mass spectrometry (MS) overcomes chromatographic delays. However, the analysis of *n*-alkane mixtures by MS is difficult because many fragment ions are formed, which leads to overlapping signals of the homologous series. Atmospheric pressure chemical ionization (APCI) is suitable for the analysis of saturated hydrocarbons and is the subject of current research. Still, although APCI is a “soft ionization” technique, fragmentation is typically inevitable. Moreover, it is usually applied for liquid samples, while an application for online gas-phase monitoring is widely unexplored. Here, we present an automated APCI-MS method for an online gas-phase analysis of volatile and semi-volatile *n*-alkanes. Mass spectra for *n*-heptane and *n*-decane reveal [M-H]^+^, [M-3H]^+^ and [M-3H+H_2_O]^+^ as abundant ions. While [M-H]^+^ and [M-3H]^+^ show an excessive fragmentation pattern to smaller C_*n*_H_2*n*+1_^+^ and C_*n*_H_2*n*-1_^+^ cations, [M-3H+H_2_O]^+^ is the only relevant signal within the C_*n*_H_2*n*+1_O^+^ ion group, i.e., no chain cleavage is observed. This makes [M-3H+H_2_O]^+^ an analyte-specific ion that is suitable for the quantification of *n*-alkane mixtures. A calibration confirms the linearity of C_7_ and C_10_ signals up to concentrations of ~1000–1500 ppm. Moreover, validated concentration profiles are measured for a binary C_7_/C_10_ mixture and a five-alkane C_7_/C_10_/C_12_/C_14_/C_20_ mixture. Compared to the 40-min sampling interval of the reference gas chromatograph, MS sampling is performed within 5 min and allows dynamic changes to be monitored.

**Graphical Abstract:**

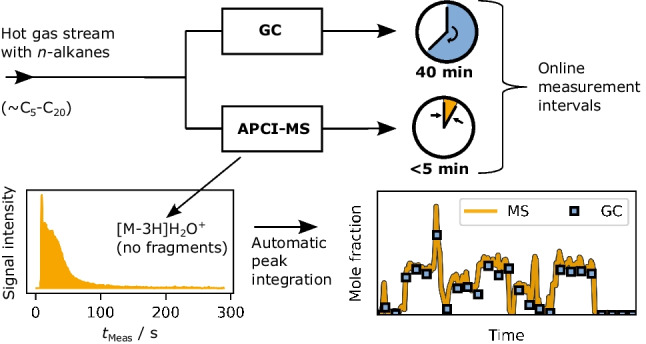

**Supplementary Information:**

The online version contains supplementary material available at 10.1007/s00216-024-05182-3.

## Introduction

In the chemical industry, a reliable analytical instrumentation is indispensable for enabling suitable process control strategies and ensuring good product qualities, from economic and/or ecological perspectives [1–5]. Modern processes make use of automated, online, real-time, or even *in-situ/operando* analytical measurements that are capable of monitoring the product’s composition over time and provide the opportunity for direct interventions [6]. Moreover, rapid analytical procedures allow for investigating dynamic process conditions and thus reveal temporal phenomena, e.g., in chemical reactors. Dynamic conditions may originate from the ongoing process itself (e.g., changing reaction kinetics, residence time distributions, transient phase changes) or be externally forced by intentional dynamic operation. For instance, dynamic operation of catalytic reactors is gaining an increased interest, as it allows to handle a varying feedstock supply (e.g., in power-to-X technologies with renewable energy fluctuations [7–10]) or provides opportunities of process intensification [11–13].

Processes that are relevant in the fuel and chemical production industry, such as petrochemical processes, biomass/waste pyrolysis or Fischer-Tropsch synthesis, can produce a wide range of volatile organic compounds (VOCs) semi-volatile organic compounds (SVOCs) in the gas phase [14–16]. The most common analytical strategy for an online gas-phase analysis of VOCs and SVOCs implies gas chromatography (GC), where even complex hydrocarbon mixtures can be separated and analyzed species-wise. However, GC requires a rather long measuring time, especially when the sample contains a wide range of hydrocarbons.

To speed up online gas-phase analysis, analytical devices with high scan rates can be used, for instance via infrared spectroscopy (IR) or mass spectrometry (MS). However, for hydrocarbon mixtures with lacking functional groups, the resulting spectra consist of overlaying signals, which are difficult to interpret. Especially mass spectrometry has been regarded as a promising tool for hydrocarbon analysis, because its versatility provides a full repository of analytical techniques [17]. Many options for sample ionization exist, including “soft ionization” techniques that aim to preserve the molecular identity of the analyte. Moreover, nowadays available ultrahigh-resolution devices (Fourier transform ion cyclotron resonance (FT-ICR) or *Orbitrap*) are capable of resolving structure and elemental composition even of complex petroleum mixtures [18, 19]. For hydrocarbon gas-phase monitoring, particularly proton transfer reaction (PTR)-MS [20–22] as well as photon ionization (PI)-MS [23, 24] has gained a lot of interest. However, many online gas-phase MS studies focus on aromatic, unsaturated, and/or heteroatomic molecules, since saturated straight-chain hydrocarbons, i.e., *n*-alkanes, are generally more reluctant to ionization and are therefore difficult to analyze [22, 25, 26]. Some MS studies successfully monitor gas-phase *n*-alkanes, e.g., by carefully choosing a suitable reagent molecule during chemical ionization (CI)-MS [26–28] or by applying single photon ionization (SPI)-MS using vacuum ultra-violet radiation (VUV) [29–32]. However, finding suitable, widely applicable and robust online methods for *n*-alkane characterization is still an ongoing research topic.

Atmospheric pressure chemical ionization, utilized in positive ionization mode (APCI(+)), is regarded as suitable ionization technique to measure saturated hydrocarbons, by forming [M-H]^+^, [M-3H]^+^, or M^+•^ ions as well as nitrogen and/or oxygen-containing adducts [33, 34]. During APCI, a corona discharge needle is used to form reagent ions, which further ionize the analyte molecules [35]. Typical reagent ions are O_2_^+•^, NO^+^, N_2_^+•^, N_2_H^+^ as well as H_3_O^+^; the latter occurs due to trace amounts of water vapor in the air [36, 37]. In gas-phase or aerosol monitoring applications, APCI(+)-MS is regarded as an alternative option for VOC analysis, again usually applied for unsaturated, aromatic, and/or heteroatomic compounds [38–40].

With respect to *n*-alkanes, most APCI(+)-MS investigations deal with liquid analytes, ranging from VOCs up to heavy petroleum species. Here, a solvent is used, which—after vaporization and ionization—usually serves as the source of the subsequent reagent ion itself [41]. Generally, any formed ion may be part of subsequent ionization processes. Unfortunately, in most cases, many fragment ions are formed, which may lead to overlapping signals of different analytes [42, 43]. Thus, the APCI ionization process is highly complex and there is ongoing research in observing and controlling APCI spectra of saturated hydrocarbons. For instance, Marotta and Paradisi studied the analysis of C_5_–C_8_ alkanes and identified several routes of how [M-H]^+^ ions may be formed in air plasma, including the reagent gases O_2_^+•^, NO^+^, H_3_O^+^, and hydrocarbon fragments [34]. Owen et al. measured lignin model compounds as well as *n*-hentriacontane (C_31_H_64_) and showed that ion distribution can be simplified by using CS_2_ as solvent and nitrogen as source gas, which led to abundant M^+•^ ions and only minor fragmentation [44]. Gao et al. reported no or only little fragmentation and [M-H]^+^ as the only significant ion for *n*-hentriacontane when using pentane or hexane as solvent and nitrogen as source gas [41]. Hourani and Kuhnert investigated a direct infusion analysis of light shredder waste samples in heptane using APCI with N_2_ as source gas and enabled a quantification in the range of C_7_–C_40_ using [M-H]^+^ [25]. Tose et al. investigated the influence of different solvents for analyzing saturated, unsaturated, and oxygen-containing compounds in paraffin samples and reported iso-octane to be favorable for the first class and pentane for the latter ones with nitrogen as ion source gas [45]. Nyadong et al. coupled APCI with laser-induced acoustic desorption (LIAD) and applied both N_2_ and O_2_ as reagent gas. A C_21_–C_40_ alkane mixture as well as a paraffin mixture were analyzed. With N_2_, predominantly M^+•^ ions were formed including significant fragmentation, while using O_2_ led to mainly [M-H]^+^ ions with only minor fragmentation [46]. A benefit of using O_2_ as ion source gas was confirmed by Jin et al. and Manheim et al. who analyzed lubricant oils by APCI-MS and determined stable [M-H]^+^ signals [35, 47]. An influence of the three ion source gases N_2_, He, and synthetic air on the ionization of saturated hydrocarbons (C_5_–C_102_) was evaluated by Souza et al., where helium and synthetic air showed better ionization efficiencies than nitrogen [48]. Again, [M-H]^+^ was the dominant ion. Recently, Li et al. characterized heavy base oils by APCI-MS and compared results with GC×GC/EI-MS evaluation. [M-H]^+^ ions were again produced for alkane species, but analysis of smaller alkanes failed due to an overlap with fragment ions. Interestingly, O_2_ and N_2_ as ion source gases gave identical results [43].

While most of the publications suggested hydride abstraction as the responsible mechanism for forming [M-H]^+^ ions, Manheim et al. determined this assumption to be wrong [42]. Instead, the authors concluded that primarily formed strong Brønsted acids, e.g., N_2_H^+^ and H_3_O^+^, transfer a proton to saturated hydrocarbons, which form [M-H]^+^ ions via subsequent H_2_ elimination. As the proton transfer is highly exothermic, the authors regarded it as the reason for fragmentation.

The literature studies highlight that there are exemplary successful applications of APCI(+) for analyzing saturated hydrocarbons (single compounds as well as hydrocarbon mixtures), often without chromatographic pre-separation. However, detailed ionization and fragmentation mechanisms and hence a reliable spectrum interpretation are still under discussion.

Although APCI-MS of gaseous hydrocarbons is not new, the method is usually applied for liquid samples. An online gas-phase monitoring application of volatile and semi-volatile *n*-alkanes via APCI(+) has, to the best of our knowledge, not been addressed in literature so far. As liquid samples are vaporized anyway, a direct gas-phase application can be assumed to be feasible and faster than conventional GC measurements.

In this work, we present a fully automated APCI(+)-MS method as a tool for a rapid online gas-phase analysis of *n*-alkanes. Measured spectra, most abundant ion groups, and fragmentation patterns are systematically investigated for *n*-heptane and *n*-decane as exemplary alkanes, to identify the most promising ion type that is suitable for a quantification in *n*-alkane mixtures. We further show that species calibration is feasible and applicable to both a binary and a five-component alkane mixture. By comparing temporal concentration profiles to a state-of-the-art GC system, the potential of increasing the analysis frequency via APCI(+)-MS is demonstrated.

## Materials and methods

### Experimental setup

**Fig. 1:**
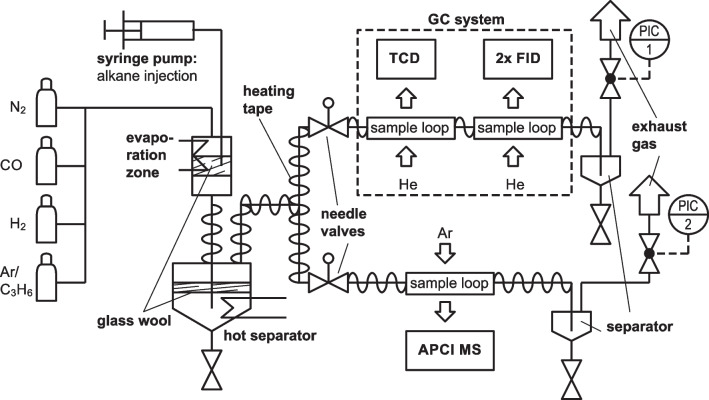
Experimental setup for investigating hydrocarbon standards by online APCI(+)-MS; the independently performed GC-FID measurements serve as a reference

The experiments were performed in a lab-scale evaporation set-up according to Fig. 1. *n*-heptane (VWR chemicals, 99.9% purity) and *n*-decane (Thermo Scientific, 99% purity) as well as a mixture of those two served as alkane standards. In a subsequent five-component experiment, *n*-dodecane (VWR chemicals, ≥99% purity), *n*-tetradecane (Alfa Aesar, 99% purity), and* n*-eicosane (SigmaAldrich, ≥99.8% purity) were added as well. The liquid is injected via a syringe pump, equipped with a pressure-tight stainless-steel syringe (both KD Scientific), into a heated tube, where the liquid is vaporized. A glass wool filling at the injector tip served as a disperser for more homogenous evaporation. Vaporized gases were transported by a diluted syngas mixture (CO/H_2_/N_2_=1:2:1, 200 ml_STP_/min) as well as an internal GC standard (Ar/cyclopropane=99:1, 25 ml_STP_/min) through a separator to an analytical set-up consisting of a Q Exactive Plus *Orbitrap* (Thermo Fisher) mass spectrometer and a three-channel gas chromatograph (Agilent 8860) equipped with one TCD and two FIDs. The separator, all pipes, and fittings were heated to at least 200 °C so that no condensation occurred within the periphery.

Alkane injections were performed by a programmed syringe pump program including five flow rate steps (3/2/1/0.5/0.25 µL/min), holding each flow rate for 4 h. To overcome initial evaporation inertia, especially at low syringe pump flow rates, it was advantageous to go from high to low injection speeds. The calibration experiments using *n*-heptane or *n*-decane only were repeated three times.

It is important to emphasize that both analytical devices were not combined as found in conventional GC/MS applications, but installed in parallel, i.e., they worked completely independently. While the GC device already had pre-installed injection valves and two sample loops (500 µl) for TCD and FIDs respectively, an additional temperature-stable 6-way injection valve (Vici Valco) including a sample loop (500 µl) was mounted upstream of the mass spectrometer. Hence, in contrast to often found real-time analysis applications [37, 39], the gas flow was not guided directly into the ionization chamber, but sampled via a valve switch and a constant argon carrier gas flow. In this way, a reproducible and process-independent sample injection was ensured. With no GC or MS method running, all sample loops were permanently purged by the gas flow. Two needle valves ensured an equal mass flow towards both instruments. The sample loop outflow was guided to individual separators at room temperature to collect condensing hydrocarbons. Pressure was controlled by two regulators and the end of the set-up to a relative pressure of 0.3 bar to facilitate sampling of the condensate.

The gas chromatograph served as a reference instrument to validate and calibrate the MS results. The GC method was a state-of-the-art analytical procedure to detect hydrocarbon species in the range from C_1_ to C_20_. For quantification, the calibrated internal standard cyclopropane (C_3_H_6_) was used. As the weight-specific FID response factor of all *n*-alkanes is nearly equal to one [49], we calculated the mole fraction of any *n*-alkane by

1 $${y}_{{{\text{C}}}_{i,\mathrm{ GC}}}={y}_{{{\text{C}}}_{3}{{\text{H}}}_{6}}\bullet \frac{{A}_{{{\text{C}}}_{i}}}{{A}_{{{\text{C}}}_{3}{{\text{H}}}_{6}}}\bullet \frac{{M}_{{{\text{C}}}_{3}{{\text{H}}}_{6}}}{{M}_{{{\text{C}}}_{i}}},$$

where ${y}_{{{\text{C}}}_{3}{{\text{H}}}_{6}}$ is the known mole fraction of cyclopropane, ${A}_{{{\text{C}}}_{i}}$ and ${M}_{{{\text{C}}}_{i}}$ are the peak area and the molar mass of the *n*-alkane analyte with the chain length *i*, and ${y}_{{{\text{C}}}_{3}{{\text{H}}}_{6}}$, ${A}_{{{\text{C}}}_{3}{{\text{H}}}_{6}}$, and ${M}_{{{\text{C}}}_{3}{{\text{H}}}_{6}}$ are the known mole fraction, the peak area, and the molar mass of cyclopropane. The GC measurement time was 33.5 min, allowing to capture alkanes up to C_20_. When only *n*-heptane or *n*-decane were investigated, the method duration could be reduced to 10 min or 17 min, respectively. More details about the GC configuration are given in the Supplementary Information.

### APCI-MS method

The mass spectrometer was equipped with its APCI ion source, utilized in positive ionization mode (APCI(+)) over a mass-to-charge range of *m*/*z* 50–350. Mass-to-charge ratios below 50 could not be detected by the device, as it is usually applied for analyzing liquid samples. Hence, the gas components CO, N_2_, H_2_, and Ar as well as low-mass hydrocarbon fragments could not be analyzed. The presented method is therefore limited in the detection of low-mass VOCs and focuses on a liquid fuel range (~C_5_–C_20_ alkanes). To prevent species condensation, the vaporizer temperature was set to 400 °C. The capillary temperature was found to have a significant influence on the magnitude of fragmentation and was therefore set to only 250 °C. All MS parameters are listed in Table 1.

**Table 1:** Applied MS parameters

MS parameter	Value
Scan Mode	Full scan: *m/z* 50–350
Resolution	70,000
Microscans	3
AGC target	5×10^6^
Max. injection time	20 ms
Sheath gas (N_2_)	2 (arb. units)
Aux gas (N_2_)	0 (arb. units)
Sweep gas (N_2_)	10 (arb. units)
Vaporizer temperature	400 °C
Capillary temperature	250 °C
Corona discharge current	200 nA

The sample loop injection was fully automated and executed by the MS method itself (via *external circuit* output). With a mass resolution of 70,000 and three microscans, the scan rate was about 1 scan/s. The signals of the ionized hydrocarbon gases were integrated to a peak area, determining the species concentration. The total measurement duration was selected to be 289.8 s (=4.83 min) in order to capture peak tailing. To enable an online monitoring, we did not evaluate the data using the standard *Xcalibur (Thermo Fisher)* software, but implemented an automatic workflow for processing the .raw files using a Python script. In this workflow, a list of all mass-to-charge ratios and its intensities of every scan is produced and anticipated ions are looked up within a defined *m/z* tolerance (measured *m*/*z* values differ slightly from theoretic values by Δ*m*/*z* ±0.002-0.008). This way, all relevant alkane peak areas are calculated fully automated and plotted online. More details on the injection procedure and the data evaluation workflow are given in the Supplementary Information.

## Results and discussion

### APCI spectra

**Fig. 2:**
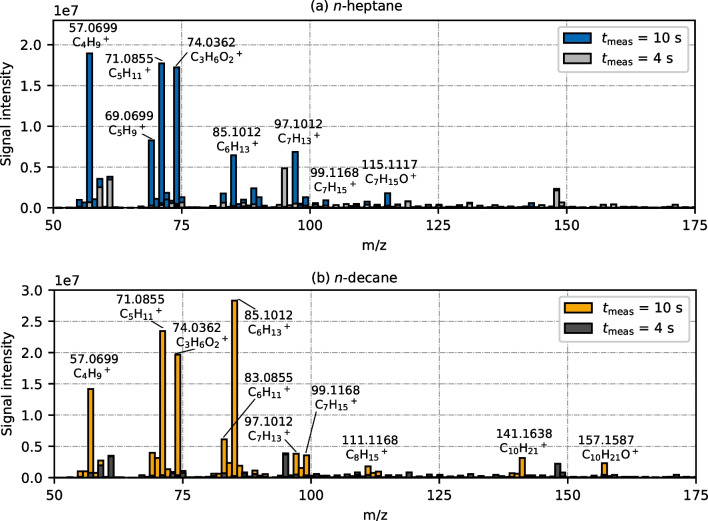
APCI spectra of the gas phase using **a** *n*-heptane and **b** *n*-decane as analyte. Signals are presented at a measurement time of ${t}_{{\text{meas}}}=10 ~ {\text{s}}$. The gray bars show background signals that are present before sample injection (${t}_{{\text{meas}}}=4 ~ {\text{s}}$)

Every MS sample injection period consisted of almost 300 spectra. Despite the fact that cyclopropane has a molecular mass below 50 u, it showed a characteristic signal at *m*/*z* 74.0362, which corresponds to C_3_H_6_O_2_^+•^ (Figure S5 in Supplementary Information). All other abundant signals were related to the two analytes *n*-heptane or *n*-decane. Exemplary mass spectra of the gas mixture containing *n*-heptane and *n*-decane, respectively, are shown in Fig. 2a and b. The signals refer to a measurement time ${t}_{{\text{meas}}}=10 ~ {\text{s}}$, i.e., 4 s after sample injection. A background spectrum that evolves before sample injection (${t}_{{\text{meas}}}=4 ~ {\text{s}}$), i.e., during a pure argon stream, is added to the graphs as well. Although we measured up to *m*/*z* 350, only *m*/*z* 50–175 is depicted here, as *n*-heptane and *n*-decane did not produce significant signals in the upper mass range.

Generally, we observe a high amount of fragment ions. Most abundant signals occur for C_4_, C_5_, and C_6_ hydrocarbon chain lengths. Smaller fragments are not visible (*m*/*z* < 50). Among the fragments, alkyl cations, i.e., C_*n*_H_2*n*+1_^+^, as well as alkenyl cations (alkyl cations with additional H_2_ elimination), i.e., C_*n*_H_2*n*-1_^+^, are dominant, which is typical for APCI(+) ionization of saturated hydrocarbons [25, 33, 34]. Ions without C-C bond cleavage, i.e., [M-H]^+^ (*m*/*z* 99.1168 for *n*-heptane; *m*/*z* 141.1638 for *n*-decane) and [M-3H]^+^ (*m*/*z* 97.1012 for *n*-heptane; *m*/*z* 139.1481 for *n*-decane) were detected as well, but with smaller intensities compared to short-chained fragments. Moreover, [M-H+O]^+^ cations were measured (*m*/*z* 115.1117 for *n*-heptane; *m*/*z* 157.1587 for *n*-decane). These oxygen-containing ions are often denoted as monohydrated [M-3H+H_2_O]^+^ ions, assuming a water cluster formation based on atmospheric humidity in the ionization chamber [25, 33, 46, 50]. Although we did not verify a water-based mechanism in this study, we assumed the same formation pathway and adopted this formulation in the following evaluation.

### Peak shapes

**Fig. 3:**
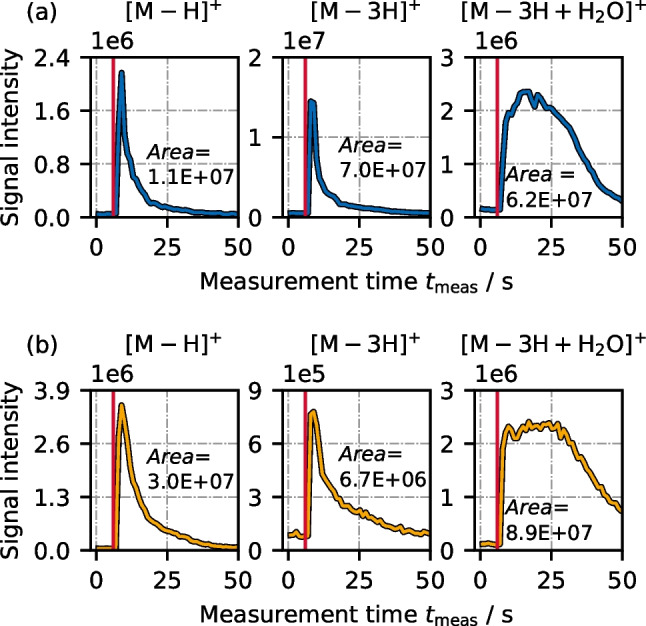
Comparison of peaks during one sample injection of [M-H]^+^, [M-3H]^+^, and [M-3H+H_2_O]^+^ for **a** *n*-heptane (${y}_{{{\text{C}}}_{7}}\approx 1500 ~ \mathrm{ ppm}$) and **b** *n*-decane (${y}_{{{\text{C}}}_{10}}\approx 900 ~ \mathrm{ ppm}$) as analyte. The vertical red line indicates the moment of sample injection

As outlined in the “APCI-MS method” section, the abundance of an ion is evaluated by integrating its signal over the measurement time, which results in a peak area. Exemplary peaks of three ions [M-H]^+^, [M-3H]^+^ and [M-3H+H_2_O]^+^ are depicted for *n*-heptane and for *n*-decane in Fig. 3a and b, respectively (at ${y}_{{{\text{C}}}_{7}{{\text{H}}}_{16}}\approx 1500 ~ {\text{ppm}},{y}_{{{\text{C}}}_{10}{{\text{H}}}_{22}}\approx 900 ~ {\text{ppm}}$). To make the most interesting part of the peaks more visible, they are only shown in the range 0 < ${t}_{{\text{meas}}}$ < 50 s.

It can be seen that [M-H]^+^ and [M-3H]^+^ had rather similar peak shapes, whereas [M-3H+H_2_O]^+^ formed a very broad peak. While the baseline value was reached within the 50 s window for [M-H]^+^ and [M-3H]^+^, tailing was more pronounced for [M-3H+H_2_O]^+^, especially with *n*-decane as analyte. This means that if only [M-H]^+^ and [M-3H]^+^ ions were to be evaluated, the measured duration could be shortened to about 1 min. However, we wanted to include the oxygen-containing ions into the evaluation and therefore chose a rather long measurement duration of about 5 min. The observation suggests that water addition occurred not immediately, but via a secondary reaction step. As metal surfaces are known to be covered by water molecules [51, 52], this secondary reaction might have occurred on the walls of the ionization chamber and hence cause a temporal delay.

Although having a lower concentration level, the *n*-decane peak areas were larger than *n*-heptane peak areas, except for [M-3H]^+^. This was expected, since the higher proton affinity of longer *n*-alkane chains should cause a higher ionization probability by proton transfer [53, 54].

### Fragmentation pattern of C_***n***_H_2***n***+1_^+^, C_***n***_H_2***n***-1_^+^, and C_***n***_H_2***n***+1_O^+^ ion groups

The fragmentation pattern is crucial for the ability of analyzing not only pure analytes but also alkane mixtures. Thus, we did not only investigate [M-H]^+^, [M-3H]^+^ and [M-3H+H_2_O]^+^ ions but all smaller fragment ions as well. We defined the three chain-length independent ion groups C_*n*_H_2*n*+1_^+^, C_*n*_H_2*n*-1_^+^, and C_*n*_H_2*n*+1_O^+^ for a detailed evaluation.

**Fig. 4:**
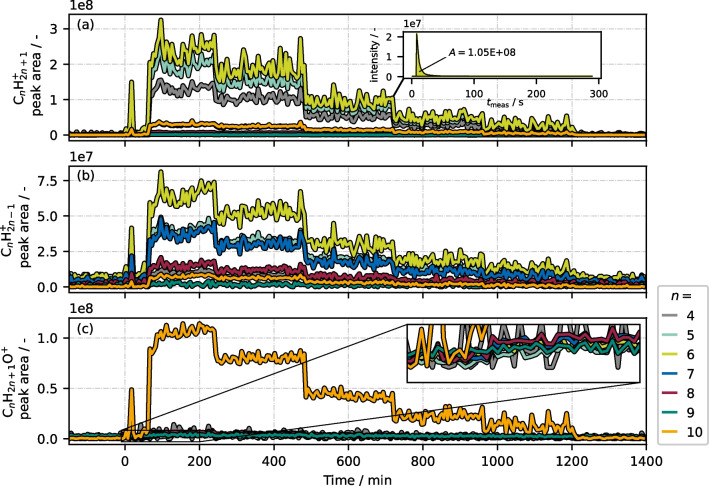
MS peak areas of the three main ion groups **a** C_*n*_H_2*n*+1_^+^, **b** C_*n*_H_2*n*-1_^+^, and **c** C_*n*_H_2*n*+1_O^+^ during a five-step experiment with *n*-decane as analyte, showing all chain lengths from *n*=4 to *n*=10. The inset in (**a**) shows a peak of the sample injection at the indicated moment. Its peak area yields in the data point in the base graph. The second inset in (**c**) shows a magnification of the marked range

The temporal development of calculated peak areas from a five-step syringe injection experiment of *n*-decane is shown for all ion groups and different chain lengths in Fig. 4.

To highlight the general data processing workflow of measured MS signals, the inset in Fig. 4a presents one exemplary peak (C_*n*_H_2*n*+1_^+^ with *n*=6), measured from one sample injection. The general signal trend is similar for all three ion groups. As fragment allocation is trivial with a single *n*-alkane analyte, each ion group is able to qualitatively depict the five concentration levels of *n*-decane. However, apparently, the fragmentation pattern is significantly different for each ion group: C_*n*_H_2*n*+1_^+^ and C_*n*_H_2*n*-1_^+^ chains are highly fragmented; for C_*n*_H_2*n*+1_^+^, especially *n*=4, *n*=5, and *n*=6 are dominant, while for C_*n*_H_2*n*-1_^+^, *n*=6, *n*=7, and *n*=8 are abundant. The ions without chain cleavage, i.e., [M-H]^+^ and [M-3H]^+^ (*n*=10), are visible but with rather low abundance. In contrast, the abundance of [M-3H+H_2_O]^+^ is not only high, but within its ion group C_*n*_H_2*n*+1_O^+^, it is the only directly observable signal. By magnifying the time period of the start of the syringe pump injection (inset in Fig. 4c), a little increment of the short fragment peak areas is indeed existent, but hardly recognizable. This fragmentation behavior, i.e., a high probability for C-C bond cleavage in case of C_*n*_H_2*n*+1_^+^ and C_n_H_2n-1_^+^, but an insignificant one for C_*n*_H_2*n*+1_O^+^ was not only observed with *n*-decane as analyte, but with *n*-heptane as well (Figure S6 in Supplementary Information). If the assumption of a secondary surface reaction with adsorbed water is correct, the low fragmentation tendency might be explained by a release of internal energy to the wall. A mechanistic investigation on the formation of [M-3H+H_2_O]^+^ is currently in progress and will be dealt with in an upcoming study. Regardless of the mechanism of formation of these ions, we were able to show that they are analyte-specific signals, which enable substance identification in *n*-alkane mixtures. Therefore, we focused on this ion type in the following evaluation and — in contrast to most previous APCI studies dealing with *n*-alkanes — neglected all other ions, such as [M-H]^+^ or [M-3H]^+^.

### Calibration of [M-3H+H_2_O]^+^ ions

**Fig. 5:**
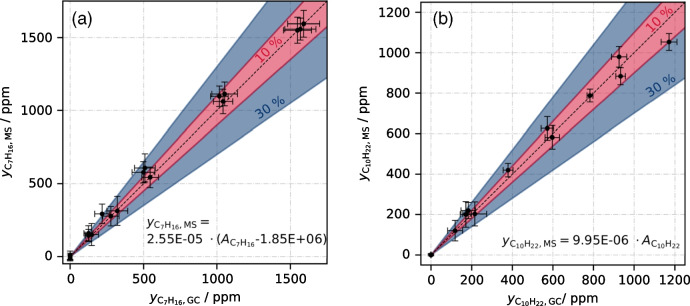
Parity plots between measured GC mole fractions and MS mole fractions, based on [M-3H+H_2_O]^+^, for **a** *n*-heptane and **b** *n*-decane. The determined [M-3H+H_2_O]^+^ calibration functions are added to the graphs as well as a 10 % and a 30 % error range

The [M-3H+H_2_O]^+^ peak area data were calibrated by scaling them to the mole fractions measured by the GC reference instrument. All small amounts of fragment ratios within the C_n_H_2n+1_O^+^ ion group, which were determined in the previous chapter, were neglected. Parity plots between the measured mole fractions ${y}_{i}$ by GC and by MS are shown for *n*-heptane and *n*-decane in Fig. 5a and b, respectively. The calibration functions for the MS peak areas ${A}_{{{\text{C}}}_{7}{{\text{H}}}_{16}}$ and ${A}_{{{\text{C}}}_{10}{{\text{H}}}_{22}}$ are added to the graphs as well. All data points were averaged in each concentration step for both GC as well as MS analysis. Error bars indicate the standard deviation for both instruments. The black dashed line presents a perfect agreement; additionally, an error range of 10 % and 30 % is indicated.

The peak areas of [M-3H+H_2_O]^+^ signals go along quite linearly with the GC reference concentrations for both *n*-heptane and *n*-decane. Most data points lie within the 10 % error region; the rest is valid with a maximum error of 30 %. Moreover, standard deviations (error bars) are quite similar for the MS signals compared to the GC data, which means that the uncertainty of the MS method is in the same range as the uncertainty of the reference method or of the entire syringe injection procedure. As can be seen from the calibration functions added to the panels, an offset had to be subtracted in case of *n*-heptane, while it could be neglected for *n*-decane. This might be due to trace impurities in the system. Although one would assume a stronger memory effect for *n*-decane, the impact of an offset signal in case of *n*-heptane could be more pronounced because the signal response itself is lower (calibration factor is about 2.5-fold compared to *n*-decane).

### Binary mixture of *n*-heptane/*n*-decane

We used a binary mixture of *n*-heptane/*n*-decane (v/v = 1:1) to check whether the calibration functions are affected by the presence of the other hydrocarbon species. It is important to mention that the rest of the gas-phase matrix (CO/H_2_/N_2_/Ar/cyclopropane) remained the same, i.e., strictly speaking, the final sample was not a binary mixture. This designation only refers to the injected liquid alkane standard. To investigate more dynamic concentration profiles, we programmed a syringe pump injection procedure with multiple step changes in varying intervals. Since GC data still served as reference, syringe pump changes were not implemented too fast, so that they could be still observed by the longer GC measurements. To enable a partial species separation of *n*-heptane and *n*-decane, the temperature of the evaporation zone and the separator were set down to 50 °C.

**Fig. 6:**
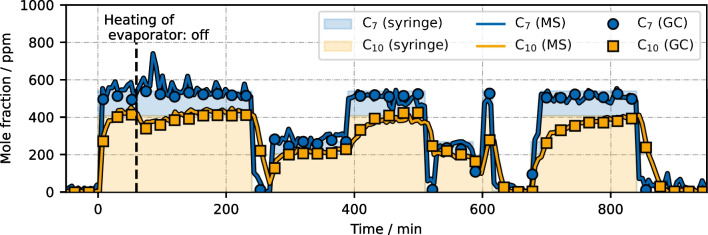
Concentration profiles during a dynamic injection experiment using a *n*-heptane/*n*-decane (v/v=1:1) mixture as hydrocarbon analyte

The mole fractions according to the syringe pump program (color-filled area) as well as the development of actual mole fractions as measured by GC and MS are presented in Fig. 6. It can be seen that the lower temperature actually led to a slower evaporation speed for *n*-decane compared to *n*-heptane. Still, both mole fractions eventually reached their expected gas-phase concentrations based on the syringe injection profile, indicating no significant remaining liquid hold-up. This behavior is consistent for all concentration changes.

The concentration profiles were well captured by both GC and MS analysis, emphasizing the suitability of the MS method to correctly measure *n*-alkane concentrations in gas-phase mixtures. It has to be noted that the MS scaling function, required for matching the GC data, slightly changed to ${y}_{{{\text{C}}}_{7}{{\text{H}}}_{16}}=2.3\times {10}^{-5}\bullet {A}_{{{\text{C}}}_{7}{{\text{H}}}_{16}}$ ; ${y}_{{{\text{C}}}_{10}{{\text{H}}}_{22}}=8.93\times {10}^{-6}\bullet {A}_{{{\text{C}}}_{10}{{\text{H}}}_{22}}$(before: ${y}_{{{\text{C}}}_{7}{{\text{H}}}_{16}}=2.55\times {10}^{-5}\bullet ({A}_{{{\text{C}}}_{7}{{\text{H}}}_{16}}-1.85\times {10}^{-6})$; ${y}_{{{\text{C}}}_{10}{{\text{H}}}_{22}}=9.95\times {10}^{-6}\bullet {A}_{{{\text{C}}}_{10}{{\text{H}}}_{22}}$). This small deviation was not due to the presence of another alkane species. The mixture experiment was not performed right after calibration and other measurements were conducted in between. A fine-tuned recalibration of the system was thus to be expected. An offset for *n*-heptane is no longer visible, which indicates that this had been an artifact due to impurities before. Although the GC measuring time was generally sufficient to capture all applied concentrations changes, mole fraction dynamics were captured in more detail by MS. Moreover, at *t* = 60 min, heating of the evaporation zone was turned off, which temporally decreased ${y}_{{{\text{C}}}_{10}{{\text{H}}}_{22}}$. A corresponding short-term increase of ${y}_{{{\text{C}}}_{7}{{\text{H}}}_{16}}$ was noticed by MS, but not by GC. This behavior cannot be verified, but it seems reasonable.

### Five-component mixture

So far, only *n*-heptane and *n*-decane were investigated as hydrocarbon analytes. To evaluate the MS method for more complex *n*-alkane mixtures, we tested a five-component *n*-alkane standard mixture consisting of C_7_/C_10_/C_12_/C_14_/C_20_ (${y}_{i}$ = 0.624/0.214/0.105/0.051/0.006). The same dynamic injection profile (Fig. 6) was used to deliver the alkane mixture into the setup. The temperature of the evaporation zone and of the separator were set to 60 °C at the start of the experiment and increased step-wise to enable some dynamic condensation/evaporation processes of the different species. The GC measurement time was increased to 33.5 min to measure hydrocarbons up to *n*-eicosane. Including the subsequent oven cooling, the interval between two GC data points was about 40 min.

**Fig. 7:**
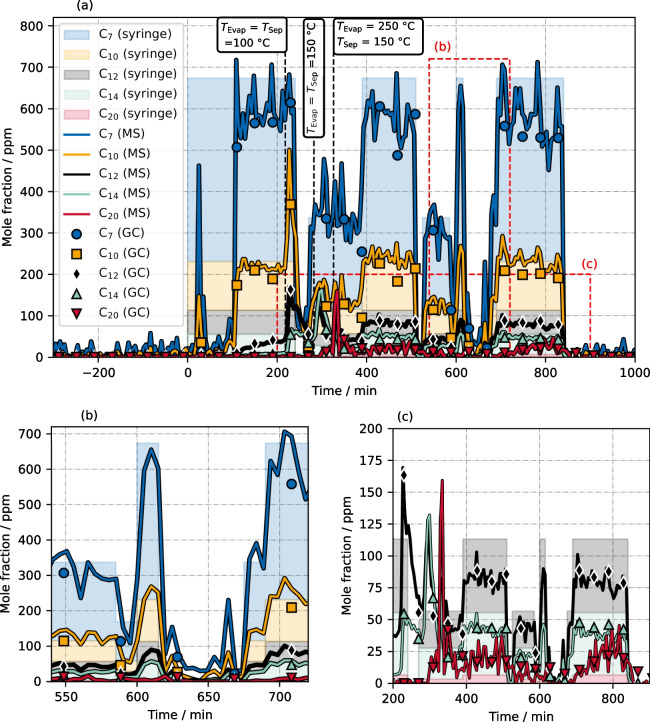
Concentration profiles during a dynamic injection experiment using a five-component *n*-alkane standard mixture consisting of C_7_/C_10_/C_12_/C_14_/C_20_ (${y}_{i}$= 0.624/0.214/0.105/0.051/0.006). Three points of temperature increase led to additional dynamic changes. The entire experiment is illustrated in (**a**); panel (**b**) and (**c**) show a magnification of the marked regions. For better clarity of the C_12_, C_14_, and C_20_ profiles, the C_7_ and C_10_ values are not shown in (**c**)

The concentration profiles measured by GC and MS are shown are Fig. 7a. Again, the theoretical syringe-injected mole fraction profiles — valid if all molecules are in the gas phase only — are added as color-filled areas. The mass-to-charge ratios (*m*/*z*) and the determined scaling parameters of [M-3H+H_2_O]^+^ ions are listed for all five components in Table 2. Here, the scaling factors for *n*-heptane and *n*-decane remained the same compared to the experiment using the binary alkane mixture. Thus, calibration is indeed independent of the other hydrocarbon analytes, which means that a simultaneous calibration of *n*-alkanes is possible.

**Table 2:** Mass-to-charge values (*m*/*z*) and scaling factors of [M-3H+H_2_O]^+^ peak areas for the five* n*-alkane components

Hydrocarbon analyte	*m*/*z* of [M-3H+H_2_O]^+^	[M-3H+H_2_O]^+^ scaling factor
*n*-heptane (C_7_H_16_)	115.1117	$2.3\times {10}^{-5}$
*n*-decane (C_10_H_22_)	157.1587	$0.893\times {10}^{-5}$
*n-*dodecane (C_12_H_26_)	185.1900	$0.3\times {10}^{-5}$
*n*-tetradecane (C_14_H_30_)	213.2213	$0.16\times {10}^{-5}$
*n*-eicosane (C_20_H_42_)	297.3152	$0.6\times {10}^{-5}$

Compared to the binary experiment, alkane species were not detected right after injection but an initial delay time is visible. We observed such a phenomenon in several experiments, indicating a saturation process of periphery surfaces. Afterwards, a different dynamic behavior of the individual species is apparent, which is due to their different vapor pressures. At the time of *t* = 200 min, *n*-heptane and *n*-decane had reached a plateau, and the signal of *n*-dodecane was slowly increasing. C_14_ and C_20_ were not yet detected. At *t* = 218 min, the temperatures ${T}_{{\text{Evap}}}$ and ${T}_{{\text{Sep}}}$ were increased to 100 °C, causing an immediate increase of ${y}_{{{\text{C}}}_{10}}$, ${y}_{{{\text{C}}}_{12}},$ and ${y}_{{{\text{C}}}_{14}}$. After the second temperature increment (${T}_{{\text{Evap}}}$ = ${T}_{{\text{Sep}}}$ = 150 °C at *t* = 282 min), ${y}_{{{\text{C}}}_{12}}$ and ${y}_{{{\text{C}}}_{14}}$ increased strongly, followed by a slow increase of ${y}_{{{\text{C}}}_{20}}$. A high mole fraction peak for *n*-eicosane was observed immediately after the third temperature increment (${T}_{{\text{Evap}}}$ = 250 °C, ${T}_{{\text{Sep}}}$ = 150 °C at *t* = 326 min). These temperature-driven concentration changes were overlapped by the dynamics caused by the syringe pump program. As expected, with increasing chain length the signal response to any applied change was slower and less intense, due to a decreasing vapor pressure.

For all five *n*-alkanes, we see a quite good agreement of GC and MS data. Hence, although not every hydrocarbon species was calibrated previously, it does not seem to be a problem to scale any *n*-alkane to the GC reference. Moreover, the results not only confirm again the suitability of the MS method to characterize hydrocarbons in gas streams, but also illustrate the benefit of an increased temporal resolution. This is especially visible in the marked magnification window shown in Fig. 7b. The short-term increase of syringe pump volume flow was not recognized by GC, but captured quite well by MS. With an MS data point interval of about 5 min, temporal resolution is increased by a factor of 8 compared to the GC measurements and is thus better suited for measuring the system dynamics.

A closer look to the concentration profile of C_20_ (second magnification window in Fig. 7c) reveals two striking aspects. Firstly, the measured mole fractions of *n*-eicosane were higher than their theoretical values as derived from the syringe pump profile, while C_12_ and C_14_ were lower (for better illustration, data of C_7_ and C_10_ are not shown here). As most C_20_ molecules accumulated as liquid within the setup after starting the experiment and evaporated only after increasing the temperature, this observation is reasonable and demonstrates the transient separation process. Secondly, although generally matching the GC data quite well, the MS data of C_20_ shows more signal fluctuations compared to the other species. Within the setup, less volatile species usually respond more slowly to temperature changes, so that the oscillations for C_20_ do not really make sense. An explanation could be a the poor C_20_ peak quality due to significant molecule transport limitations based on low volatility. Either within the sample loop or in the ionization chamber C_20_ molecules probably remained adsorbed to the surfaces for quite a long time, so that the ion signal did not fit into the 5-min measurement window (Figure S7 in the Supplementary Information). Multiple adsorption/desorption processes likely occurred on the way to the mass analyzer. This is clearly a disadvantage compared to conventional liquid MS applications where heavy compounds are dissolved in a suitable solvent and do not suffer from major transport limitations. In this gas-phase application, improvements might be possible by increasing the temperature of sample loop, vaporizer, and/or capillary. The broad peak further explains the relatively large scaling factor for *n*-eicosane listed in Table 2, where a generally decreasing factor with increased chain length is visible. With a full-range peak, calculated C_20_ peak areas would be larger, resulting in a lower response factor. Nevertheless, despite the low peak quality, the MS data of C_20_ follow the trend of GC measurements quite well, i.e., a clear correlation with the mole fraction is visible.

## Conclusion

We presented a new method for a rapid online gas-phase analysis of volatile and semi-volatile *n*-alkanes using APCI(+)-MS using [M-3H+H_2_O]^+^ ions for quantification. In contrast to [M-H]^+^ and [M-3H]^+^ ions, [M-3H+H_2_O]^+^ ions showed negligible chain cleavage, which allowed for an explicit analyte identification and a good match of concentration profiles with GC reference data for a one-, two-, and five-component alkane mixture.

In this context, [M-3H+H_2_O]^+^ ions are assumed to be formed via secondary reactions with surface adsorbed water molecules. A corresponding mechanistic study is currently being carried out. Unfortunately, [M-3H+H_2_O]^+^ ion transport to the analyzer is slow, which prolongs the measuring time to capture the long-tailing peaks. Still, the APCI-MS method has proven to be valid and faster than a conventional GC method. The temporal benefit depends on the range of hydrocarbons to be analyzed. In this study, temporal resolution for a characterization up to *n*-eicosane was improved by a factor of 8. This way, dynamic concentration changes, which were invisible to the GC system, were captured by the MS instrument.

However, with a minimum *m*/*z* 50 of the detector used, a mixture characterization below alkane chain lengths of *n*=3 is impossible. Short-chained alkanes might be problematic anyway, as their proton affinity might be too low for ionization. For long-chained molecules, such as *n*-eicosane, significant transport limitations due to low volatility became apparent, which have to be further examined. Furthermore, matrix effects have to be assessed, by other hydrocarbons (e.g., branched, unsaturated, aromatic), heteroatomic compounds (especially oxygen-containing) and by changes of the permanent gases (e.g., CO or H_2_). The latter aspect is especially important when applying the method to reaction systems such as Fischer-Tropsch synthesis, where CO and H_2_ are consumed, i.e., the matrix changes over time. Hence, there is still a range of uncertainties that need to be clarified, which will be topic of further investigations. Nevertheless, the method is already usable to identify system dynamics in transient *n*-alkane gas-liquid systems, such as residence time experiments or separation processes.

### Supplementary Information

Below is the link to the electronic supplementary material.

Supplementary file1 (DOCX 5838 KB)

## Data Availability

All data, the automated monitoring workflow, and the evaluation scripts that were used for this study are available in an online repository [55].
