# Supplementary material for: Rapid online analysis of n-alkanes in gaseous streams via APCI mass spectrometry
Source: Anal Bioanal Chem. 2024 Feb 15;416(8):1843–55. doi: 10.1007/s00216-024-05182-3 (PMC10902047; doi:10.1007/s00216-024-05182-3)
Supplement: Supplementary file 1 — Supplementary file1 (DOCX 5838 KB) [file 216_2024_5182_MOESM1_ESM.docx]

**Supplementary Information**

**Rapid online analysis of *n*-alkanes in gaseous streams via APCI mass spectrometry**

Jonas Wentrup^a,b^, Ingmar Bösing^a,b^, Thomas Dülcks^c^ , Jorg Thöming^a,b,d*^

^a^ University of Bremen, Faculty of Production Engineering, Chemical Process Engineering,
Leobener Strasse 6, 28359 Bremen Germany

^b^ University of Bremen, Center for Environmental Research and Sustainable Technology,
Postbox 330 440, 28334 Bremen, Germany

^c^ University of Bremen, FB 02, Mass Spectrometry Service Facility, Leobener Str. NW2A, 28359 Bremen, Germany

^d^ University of Bremen, MAPEX Center for Materials and Processes,
Postbox 330 440, 28334 Bremen, Germany

^*^ corresponding author (J. Thöming: [thoeming@uni-bremen.de](mailto:thoeming@uni-bremen.de))

**SI-1: Gas chromatography configuration**

The GC method was performed using an Agilent 8860 System, equipped with one thermal conductivity detector (TCD) and two flame ionization detectors (FID). A simplified column configuration is shown in Figure S1. The two sample loops were installed in a heated valve box and constantly purged by the gas stream. Once the GC method was started, both injection valves injected their sample into the column system. Pre-columns and a backflush system on the TCD side are not illustrated for simplicity.

| 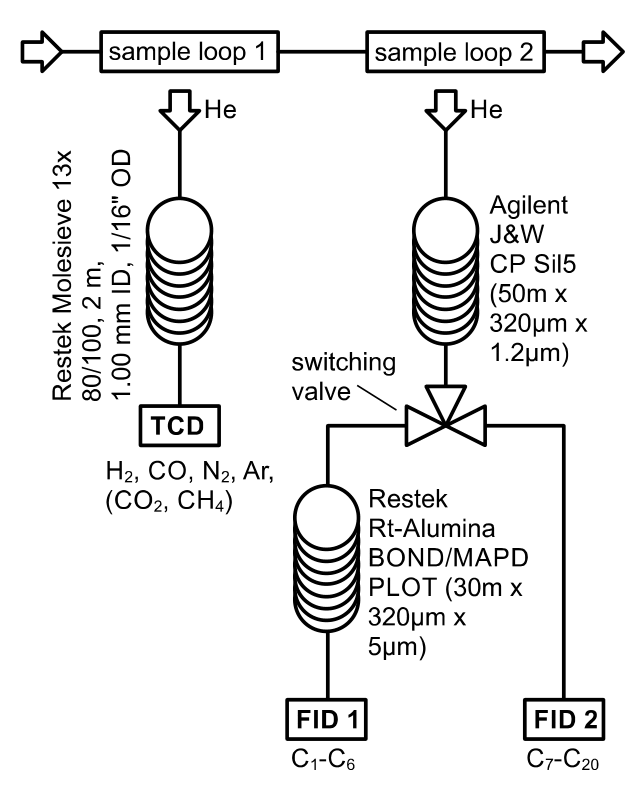 |
| --- |
| Figure S1: Simplified GC column configuration for the analysis of permanent gases as well as  C_1_-C_20_ hydrocarbons. |

The permanent gases hydrogen (H_2_), carbon monoxide (CO), nitrogen (N_2_), argon (Ar) were separated in a molecular sieve (MS-13X 80/100, 2 m, 1.00 mm ID, 1/16" OD, Restek Corporation) and detected by the TCD. Carbon dioxide (CO_2_) and methane (CH_4_) are also detectable. The hydrocarbons were split into two fractions for simultaneous analysis. Small hydrocarbons (C_1_-C_6_) are separated by a Rt-Alumina BOND/MAPD PLOT (30m × 320µm × 5µm) column (Restek Corporation), while C_7_-C_20_ hydrocarbons were separated by an CP Sil5 (50m × 320µm × 1.2µm) column (Agilent J&W). A switching valve ($t_{\mathrm{switch}}=6.5 \min$) guided the volatile hydrocarbon fraction to FID 1, while the higher fraction was held back in the CP SIL5 column and guided to FID 2. The applied temperature program started at 60 °C (6 min hold), increased to 120 °C with 10 °C/min, increased to 250 °C with 20 °C/min and was held at 250 °C for 15 min ($t_{\mathrm{total}}=33.5 \min$). If the sample contained only hydrocarbons smaller than C_20_, the method could be shortened accordingly.

**SI-2: APCI-MS injection procedure and automatic data evaluation/online monitoring**

The MS sample injection was performed using a constant argon carrier gas flow with a pressure of $p_{\mathrm{Ar}}=$5 bar. In valve position 1, the sample loop was filled with gas mixture, while argon was guided directly to the MS system. After filling the sample loop for 6 s, the valve position was switched so that an argon carrier gas flushed the sample into the ionization chamber (position 2). After another 9 s, the valve was switched back to position 1. The total measurement duration was selected to be 289.8 s (=4.83 min) in order to capture peak tailing. To make sure that the injection period of 9 s was long enough to empty the sample loop completely, we varied the moment of valve switching by several seconds in both directions without noticing any difference in the signals. The fact that peak tailing was still detected means that sample injection was faster than ionization and ion transport. Including a processing delay time between two measurements, the temporal resolution of the MS method was below 5 min. The injection procedure and a schematic signal response is illustrated in Figure S2.

| 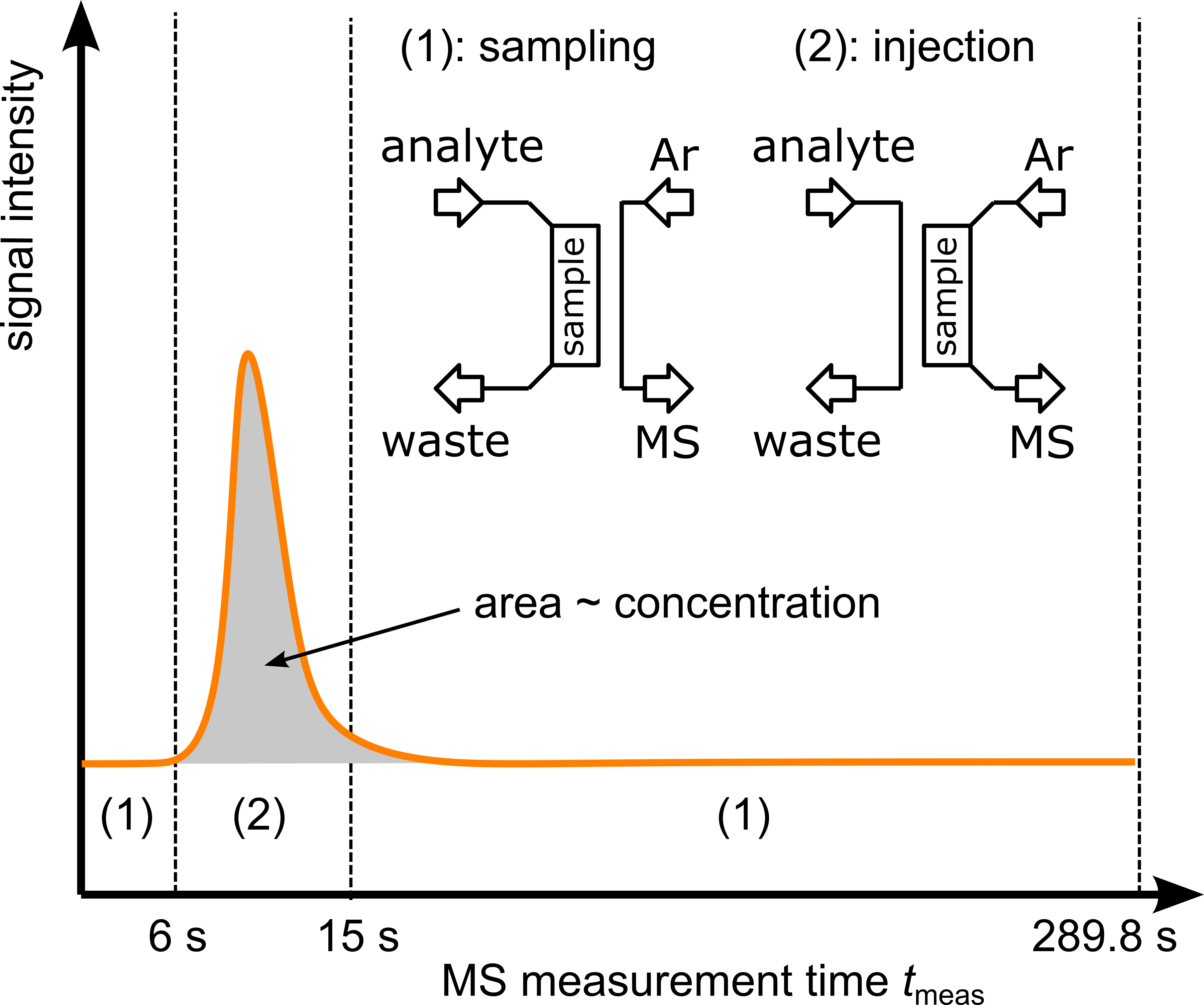 |
| --- |
| Figure S2: MS injection procedure of the two-position valve including a schematic signal response. |

To enable an online evaluation and monitoring of the APCI-MS raw data, a workflow was established which is completely decoupled from standard *Xcalibur* software quantification (Figure S3). In general, the evaluation procedure is based on the ThermoRawFileParser [1], which is available at <https://github.com/compomics/ThermoRawFileParser>. It converts the ThermoFisher RAW file format into a JSON file, which contains all spectral data of the measurement and a TXT file, which includes some further meta data. These files can be imported by a Python script using standard modules. The spectral data were used to calculate all relevant peak areas. The meta data contains the creation date and hence delivers the temporal information for the online measurement.

| 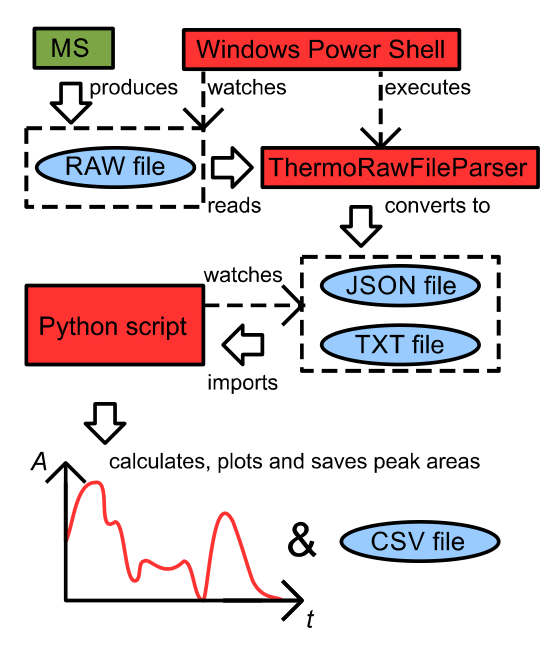 |
| --- |
| Figure S3: Schematic flow diagram of the online monitoring workflow. |

Both the RAW file parsing and the python import were automated using a directory watching code. RAW files were watched by a Windows Power Shell script. Once a new RAW file was created, the previous RAW file was read and converted by the ThermoRawFileParser. This delay of one measurement was necessary to ensure that the RAW file was completed and not changed any more. This means that the sample information is not monitored in real-time, but with a delay of one measurement cycle (= 5 min). The Python script watched the JSON and TXT files. As soon as a new parsing process was finished and these files existed, they were imported by the Python script. All relevant hydrocarbon mass-to-charge ratios were looked up and the corresponding signals were used to form respective peaks. For each mass, a baseline was formed by the mean of the first and last data points and a peak area was calculated. These data were plotted in a constantly live-updating graph and moreover saved as CSV file, to allow a quick data import for further analysis. The entire workflow is available in an online repository [2].

**SI-3: GC measurements**

Figure S4(a) and S4(b) show the measured mole fractions of *n*-heptane and *n*-decane during all three five-step injection experiments, respectively. In an additional inset graph, the mean mole fractions of each concentration step are plotted against the syringe injection volume flow.

| 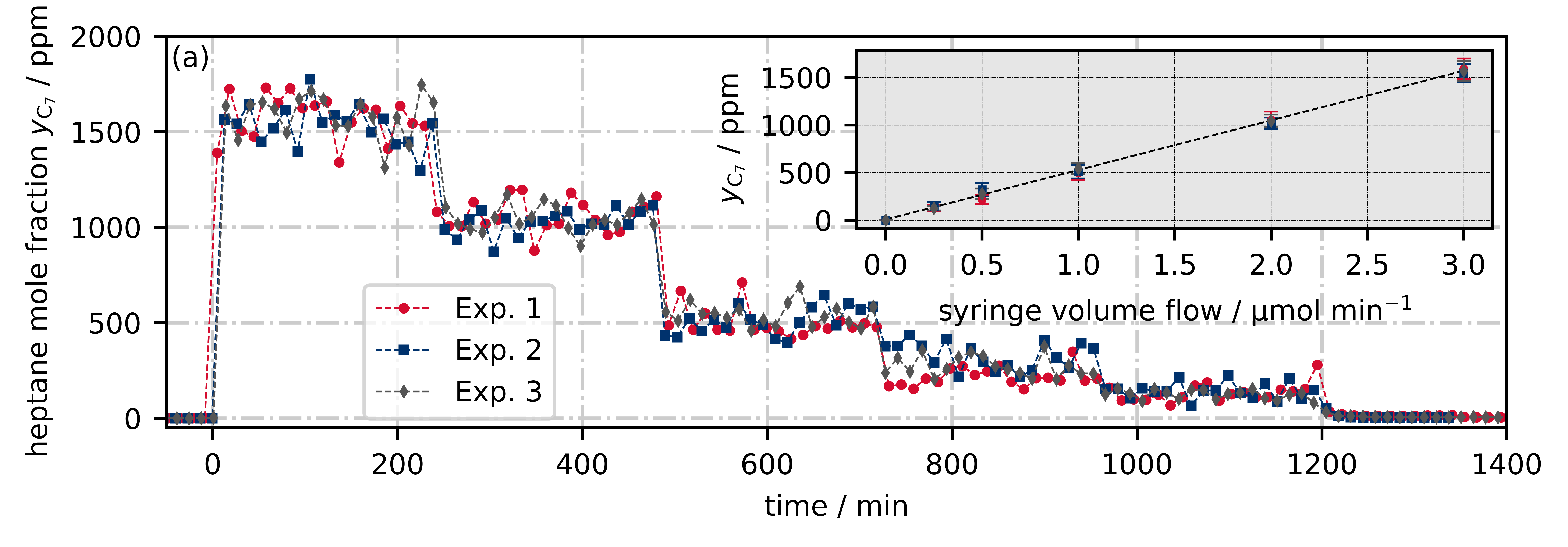 |
| --- |
| 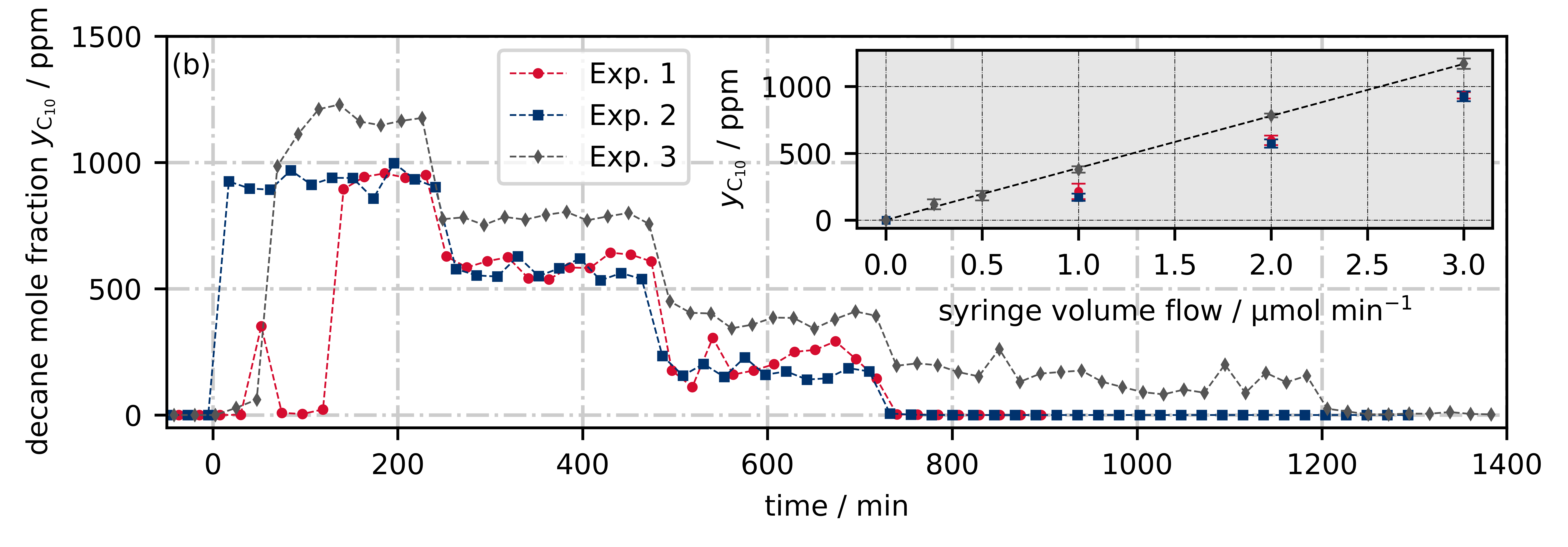 |
| Figure S4: Measured concentration profiles by the reference GC system with (a) *n*-heptane and (b) *n*-decane as injected alkane standard. |

For *n*-heptane, reproducible and linear GC measurements were produced. Both the temporal concentration profiles and the mean values differed only by small deviations (error bars in the inset indicate standard deviation). In the case of *n*-decane, the concentration profiles of the first and second experiment were quite similar, but showed only three concentration levels, and only the third experiment showed all five levels which were moreover higher than measured before. This was caused by a leakage right after the syringe originating from a non-sealed fitting. Hence, the syringe volume flow in experiment 1 and 2 was only partly guided into the set-up, leading to lower mole fraction levels and made the last two steps invisible.

**SI-4: Gas-phase matrix**

Apart from the investigated hydrocarbons, the gas-phase included a constant matrix consisting of hydrogen (H_2_), carbon monoxide (CO), nitrogen (N_2_), argon (Ar), cyclopropane (C_3_H_6_). Except for cyclopropane, none of these species showed a signal in the mass spectrum, as *m/z*<50 could not be detected (Figure S5). Cyclopropane mainly showed an ion at *m/z*=74.0362, which corresponds to C_3_H_6_O_2_^+•^, and some minor signals, e.g. *m/z*=91.0628 (C_3_H_9_O_2_N^+•^). Possible formation schemes of these ions are not further discussed in this study.

| 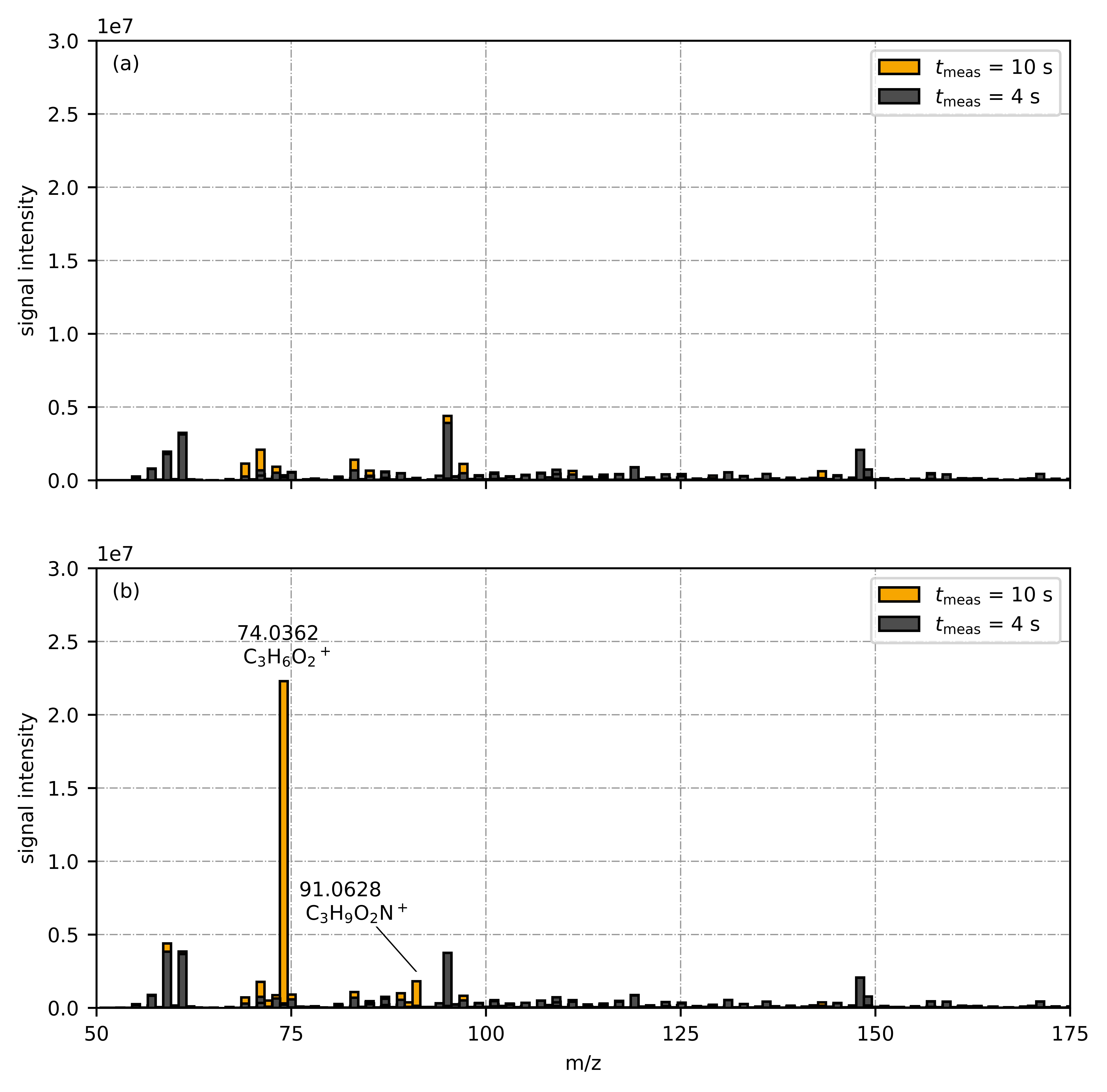 |
| --- |
| Figure S5: Mass spectrum of (a) the gas matrix H_2_/CO/N_2_/Ar (without cyclopropane) and (b) the gas matrix H_2_/CO/N_2_/Ar/C_3_H_6_ (with cyclopropane) at two different measurement times  (4 s: before sample injection, 10 s: after sample injection). |

**SI-5: Peak areas of different ion groups with *n*-heptane as analyte**

In addition to the exemplary temporal peak area development of the three different ion groups with *n*-decane as analyte (Figure 4 in manuscript), Figure S6 presents an exemplary signal course of a five-step experiment for *n*-heptane. All other experiments can examined by using the data in the online repository [2]. Similar to *n*-decane, C*_n_*H_2_*_n_*_+1_^+^ and C*_n_*H_2_*_n_*_-1_^+^ chains show a high abundance of smaller fragments, while [M-3H]H_2_O^+^ show only negligible chain cleavage. The only significant difference between *n*-heptane and *n*-decane is that [M-3H]^+^ (panel (b)) has significantly higher abundance in case of *n*-heptane. This aspect is not further discussed in this study.

| 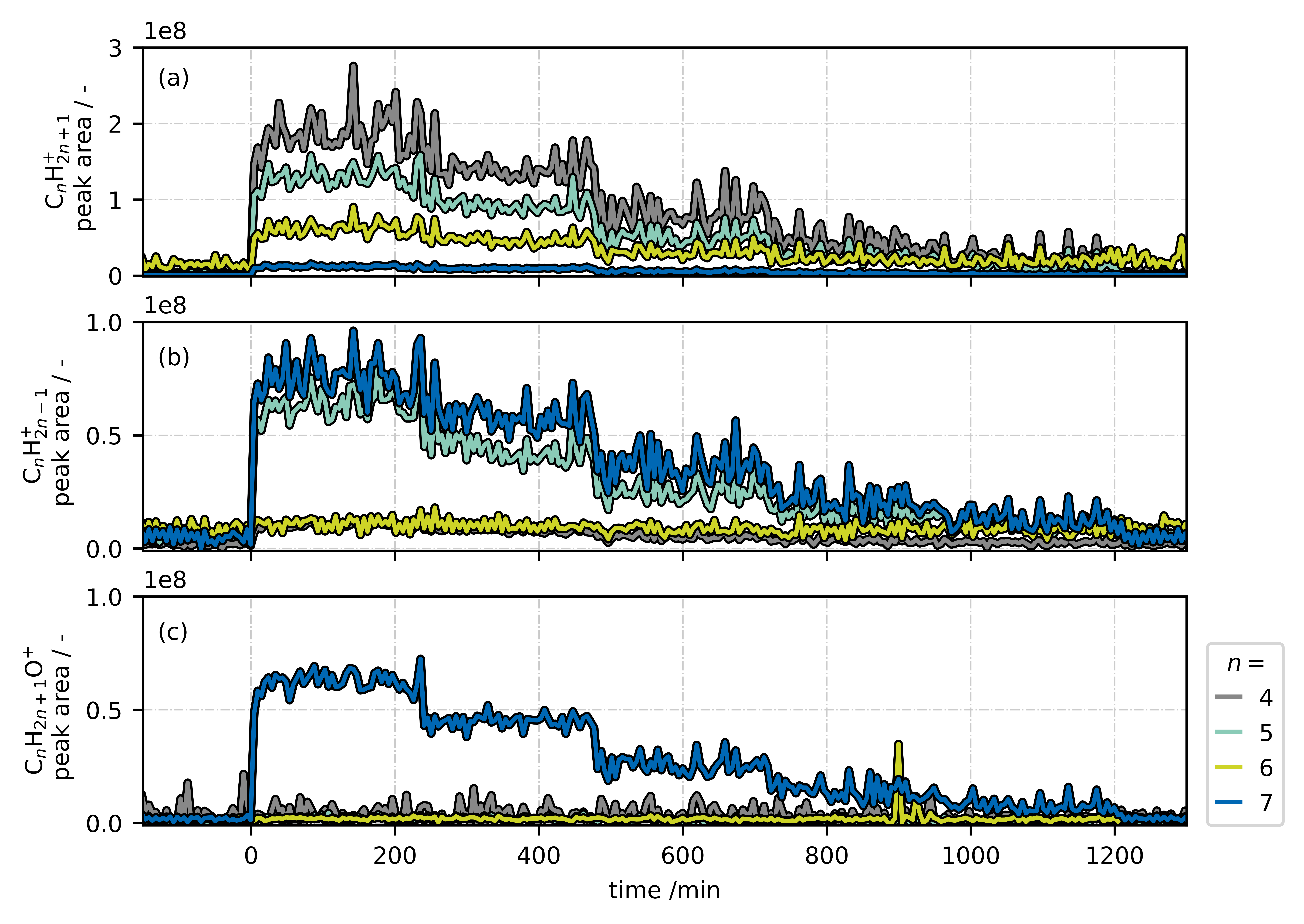 |
| --- |
| Figure S6: MS peak areas of the three main ion groups (a) C*_n_*H_2_*_n_*_+1_^+^, (b) C*_n_*H_2_*_n_*_-1_^+^ and (c) C*_n_*H_2_*_n_*_+1_O^+^ during the second experiment with *n*-heptane as analyte, showing all chain lengths from *n*=4 to *n*=7. |

**SI-6: Peaks of five-alkane mixture**

During the five-alkane experiment, a fluctuating peak area was determined for [M-3H]H_2_O^+^ of *n*-eicosane (C_20_). While C_7_, C_10_, C_12,_ and C_14_ had similar peak shapes, the peak of C_20_ was so broad that it did not fit into the five-minute measurement window (Figure S7). The signal decreased strongly for the first couple of seconds (which is caused by the rest of the previous peak) and formed a broad peak afterwards. Apparently, the low volatility of C_20_ led to mass transport limitations.

| 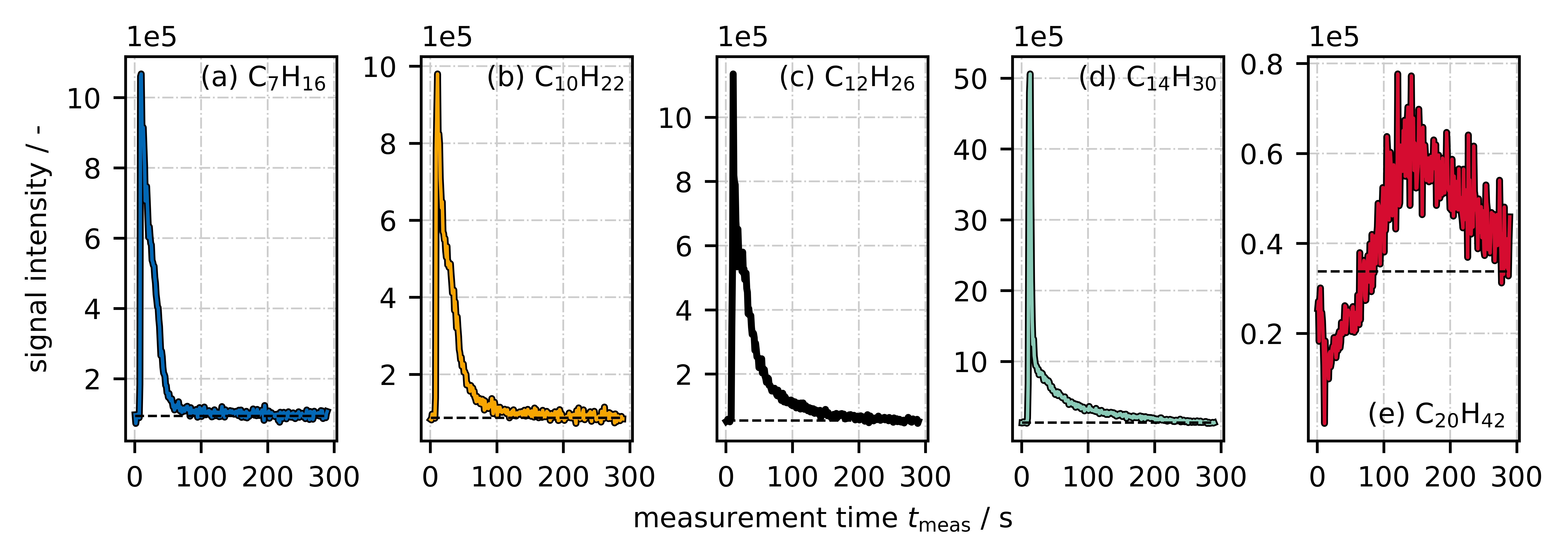 |
| --- |
| Figure S7: Signal peaks of [M-3H]H_2_O^+^ of (a) *n*-heptane, (b) *n*-decane, (c) *n*-dodecane, (d) *n*-tetradecane and (e) *n*-eicosane at *t* = 309.53 min. The dashed line presents the automatically calculated baseline. |

**References**

1. Hulstaert N, Shofstahl J, Sachsenberg T, Walzer M, Barsnes H, Martens L, Perez-Riverol Y (2020) ThermoRawFileParser: Modular, Scalable, and Cross-Platform RAW File Conversion. J Proteome Res 19:537–542. https://doi.org/10.1021/acs.jproteome.9b00328

2. Wentrup J (2023) Supporting files for “Rapid online analysis of n-alkanes in gaseous streams via APCI mass spectrometry.” https://doi.org/10.5281/zenodo.10203915
